# Commentary: Association of dietary index for gut microbiota with frailty in middle-aged and older Americans: a cross-sectional study and mediation analysis

**DOI:** 10.3389/fnut.2025.1674225

**Published:** 2025-11-20

**Authors:** Yuanfeng Han, Peishan Yu, Wenjiang Wu

**Affiliations:** Shenzhen Hospital (Futian) of Guangzhou University of Chinese Medicine, Shenzhen, Guangdong, China

**Keywords:** gut microbiota, nutritional strategies, frailty, DI-GM, commentary

## Introduction

The study's identification of a protective threshold (DI-GM ≥4.082, OR = 0.874) and subgroup effects (stronger in women/highly-educated) provides actionable public health targets. However, the tension between the title's emphasis on “gut microbiota” and the absence of microbial measurements reveals a pivotal opportunity to apply quantitative microbiome profiling (1). Can we evolve static “microbiota-linked diets” into dynamic “microbiota-responsive nutrition”—*where personalized diets are iteratively adjusted based on real-time monitoring of individual gut microbial changes*?

## Key challenges: mechanistic and translational gaps

Three interconnected constraints hinder DI-GM's current utility:

(1) *Static assumptions disregarding host-microbe dynamics*,

(2) *Oversimplified mediation analysis obscuring direct microbiota pathways*,

(3) *One-size-fits-all thresholds failing biological heterogeneity*.

The static assumptions disregard host-microbe dynamics, as age-related physiological shifts (e.g., reduced gastric acid) alter microbial responses to identical foods—a process heavily influenced by age-dependent microbiome remodeling (2). For example, only 30%−50% of individuals convert soy isoflavones to bioactive equol, directly compromising anti-inflammatory efficacy. Simultaneously, oversimplified mediation analysis obscures direct microbiota pathways; attributing 38% of effects to BMI may overlook unmeasured mechanisms like SCFAs (Short-chain fatty acids) regulating muscle synthesis via HDAC inhibition (Histone Deacetylase inhibition). Crucially, one-size-fits-all thresholds (DI-GM ≥4.082) fail in biologically diverse populations, failing individuals with baseline dysbiosis (e.g., Bacteroidetes/Firmicutes ratio < 0.8), thereby necessitating urgent precision adaptation.

**Figure 1:**
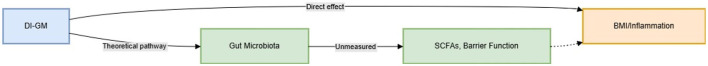
Proposed pathways linking DI-GM to frailty risk. Solid orange arrows: empirically supported direct effects of DI-GM on BMI/inflammation. Dashed blue arrows (“theoretical pathway”): hypothesized but unvalidated DI-GM effects on gut microbiota composition. Dashed green box (“unmeasured”): critical gaps in quantifying microbial mediators (*SCFAs, barrier function*).

As depicted in Figure 1, while the direct effect of DI-GM on BMI/inflammation (solid orange arrow) enjoys empirical support, the purported microbiota-mediated pathway suffers from critical discontinuities: the critical gap between DI-GM and actual gut microbiota changes (e.g., species-level abundance shifts) remains unvalidated experimentally (blue dashed arrow labeled “Theoretical pathway”), and downstream microbial mediators—specifically SCFAs and barrier function enclosed in a green “Unmeasured” dashed box—lack quantitative assessment. This dual measurement gap reduces gut microbiota to a hypothetical entity rather than a verified mediator, fundamentally undermining the framework's premise as a “microbiota-targeted dietary index.”

## New vision: host-microbe co-adaptation framework

To address these gaps, we propose a three-tiered integrative strategy: dynamic mechanism validation requires embedding temporal microbiota monitoring (e.g., weekly at-home stool tests) within cohorts to map trajectories from DI-GM foods → microbial gene expression (e.g., acetate kinase *ackA*) → host metabolites (β-hydroxybutyrate) → frailty phenotypes. The Dutch Aging Study demonstrated a 40% increase in butyrate-producers after 6-week high-fiber diets, yet with eight-fold inter-individual variation, underscoring the necessity for longitudinal surveillance. Phenotype-driven personalization necessitates customizing interventions by baseline microbial signatures (3): *Bacteroides*-dominant individuals benefit from resistant starch (activating *Bacteroides* amylases), *Firmicutes*-dominant cohorts require inulin supplementation (promoting *Bifidobacterium*), and low-diversity subjects prioritize fermented foods (rapid functional microbe introduction). Clinical translation culminates in developing “frailty prevention digital twins”—AI models following the personalized nutrition framework (4), generating real-time DI-GM adjustments from inputted gut microbiota, metabolic markers, and dietary records. Europe's JENI initiative achieved 53% higher frailty reversal rates with such interventions, validating practical implementation.

## Discussion

This pioneering work establishes DI-GM-frailty associations, but clinical translation necessitates conquering three concurrent barriers: Mechanistic specificity demands confirming gut microbiota-dependency via fecal transplant animal models (e.g., transferring microbiomes from high/low DI-GM individuals to gnotobiotic mice); Point-of-care innovation requires developing home SCFAs test strips to quantify microbial metabolite output (e.g., butyrate < 5 μmol/g predicting intervention failure); Socioeconomic integration must address food deserts limiting adherence in vulnerable populations (e.g., 60% reduced fresh produce access in low-income communities). Only through these advances can DI-GM evolve into the first “microbiota-explainable frailty prevention index,” shifting precision nutrition from theoretical paradigm to clinical reality.
